# Role of some inflammasomes in rheumatoid arthritis patients in Egypt

**DOI:** 10.1007/s11033-023-08738-1

**Published:** 2023-09-02

**Authors:** Reham Ibrahem, Mervat A. Raghip, Mamdouh M. Abdelwahed, Noha S. Amin, Esam M. Abualfadl, Nancy G. F. M. Waly

**Affiliations:** 1 Microbiology and Immunology Department, Faculty of Pharmacy, Minia University, Minya, Egypt; 2 Microbiology and Immunology Department, Faculty of Pharmacy, Sohag University, Sohag, Egypt; 3 Medical Microbiology and Immunology Department, Faculty of Medicine, Sohag University, Sohag, Egypt; 4 Rheumatology and Rehabilitation Department, Faculty of Medicine, Sohag University, Sohag, Egypt

**Keywords:** Inflammasomes, Rheumatoid arthritis, NLRC4, NLRP1, ASC, CASPASE-1

## Abstract

**Aim:**

This study aims to demonstrate the role of some inflammasomes genes: NLRC4 (the NLR family, CARD domain-containing protein 4), NLRP1 (NLR family, pyrin domain-containing 1), ASC (Apoptosis-associated speck-like protein containing a CARD), and CASPASE-1 in the pathogenesis of Rheumatoid arthritis (RA) in Egyptian population.

**Main methods:**

The expression level of NLRC4, NLRP1, ASC, and CASPASE-1 within PBMCs isolated from all RA subjects by quantitative real-time PCR. GAPDH gene was used as a reference gene. Measurement of serum level of IL-1β and IL-18 was performed using ELISA.

**Key findings:**

Results showed dysregulated inflammasomes expression that may participate in the pathogenesis of the inflammatory process of the disease.

**Significance:**

Understanding the role of inflammasomes in RA pathogenesis helps in finding promising therapy for the treatment and management of this disease.

## Introduction

Rheumatoid arthritis (RA) is a symmetric polyarticular arthritis that primarily affects the small diarthrodial joints of the hands and feet. Rheumatoid arthritis affects about 1% of the world's population [1, 2]. In addition to inflammation in the synovium, which is the joint lining, the aggressive front of tissue called pannus invades and destroys local articular structures [3, 4]. Unmanaged RA can lead to joint deterioration and impairment, as well as a lower quality of life and a higher mortality rate.

The diagnostic methods for detection include clinical evaluation, imaging, and laboratory tests. The extent of the inflammation and damage is confirmed by blood tests [5]. The detection of rheumatoid factor and/or anti-citrullinated protein antibodies, a raised C-reactive protein level, or an increased erythrocyte sedimentation rate in a patient with inflammatory arthritis confirms the diagnosis of RA [6]. Research in the area of inflammatory illnesses has begun to concentrate on inflammasomes [7].

Inflammasomes working at intracellular levels primarily promote the responses to inflammatory events [8]. Inflammasome is crucial component of the innate inflammatory response that is a cytoplasmic multiprotein complex that enables the removal of foreign pathogens or the identification of endogenous danger signals. Extreme or unusual activation of numerous diseases, including autoimmune disorders, neurological conditions, and malignancies, have been demonstrated to be influenced by inflammasomes [9]. Interleukin-1β (IL-1β) and IL-18 are the two main inflammatory cytokines that are produced by inflammasomes. Inflammasomes are classically formed by assembling three major components, an inflammasome threat sensor, an adaptor protein, and an effector enzyme [10]. An adapter protein, mostly the apoptosis-associated speck-like protein (ASC protein), and Caspase-1 make up inflammasomes. Sensor proteins of inflammasomes exhibit extraordinary variety, which enables the formation of specific complexes designed to detect different signals [11].

Inflammasomes can be categorized into “canonical inflammasomes,” such as nucleotide-oligomerization domain (NOD)–like receptors (NLR) and absent in melanoma 2 inflammasomes (AIM2) besides non-canonical inflammasomes like caspase-4, caspase-5, and caspase-11 [12]. The first discovered NLR family member to form an inflammasome complex was NLRP1 [13]. ASC is very vulnerable to caspases, which intensify inflammatory activity because they release macrophages and other inflammatory cytokines into the bloodstream and trigger immunological reactions when they interact with other molecules [14].

Inflammasome activity that is dysregulated in many inflammation-related disorders drives pathology in contrast to these beneficial roles. Inflammasomes have the ability to potently trigger host-protective responses to infection and pathology in a variety of serious human diseases. All protective and pathogenic actions of inflammasomes are supported by the process by which inflammatory caspases acquire protease activity within inflammasomes [15].

Dysfunctional inflammasomes are linked to the pathophysiology of joint diseases. Treatment and diagnosis of inflammasome-induced inflammatory illnesses like rheumatoid arthritis can be improved by understanding the process of inflammasome activation and how they contribute in physiological and pathological states [16, 17].

According to a study that looked at the gene expression level of inflammasomes in a total of 1278 RA patients. ASC, MEFV, NLRP3-FL, NLRP3-SL, and CASP1 gene expression were significantly higher compared with controls, whereas CARD8 was lower in the patients. Interleukin-18 and caspase-1 levels were markedly elevated in RA patients. This study is one of many studies that demonstrated the impact of dysfunctional inflammasomes on the pathogenesis of RA [18].

In this study, we focused on the study of gene expression of some inflammasomes genes in rheumatoid arthritis patients in Egyptian population to reveal the role of dysregulated inflammasomes in the pathogenesis of RA. In addition, the impact of genetic factor as one of the parameters that can influence the progress of the disease and the regulation of the inflammasomes was studied. Also, we studies the effect of RA medications on the expression level of inflammasomes and serum levels of IL-1β and IL-18 which are hallmarks of the inflammasome activation.

## Materials and methods

### Subjects

Sample from 77 subjects were included in the study. These samples were classified into three groups:

- **Group1 (treated cases):** 37 RA patients (30–72 years) receiving treatment (2 males and 35 females).
- **Group2 (untreated cases):** 15 RA patients (23–62 years) not receiving treatment (1 male and 14 female).
- **Group3 (control group):** 25 healthy individuals (24–53 years; 7 males and 18 females).

Cases were selected according to The American College of Rheumatology's updated classification criteria for rheumatic illnesses [19]. All subjects were free of infectious and autoimmune disorders. All subjects signed the informed consent form prior to the start of experiment. The study was approved by the “Commission on the Ethics of Scientific Research,” Faculty of Pharmacy, Minia University (HV03/2020).

### Sample processing

Seven milliliters of peripheral blood were collected from each subject in an ethylene diamine tetraacetic acid (EDTA) vial. Three milliliters of whole blood were used for RNA extraction [20], two milliliters were used for ESR detection, and the remaining two milliliters were used for serum isolation and cytokines, RF, and Anti-CCP detection**.**

### PBMC isolation from whole blood

Three milliliters of whole blood were placed over Histopaque-1077 (Sigma-Aldrich, Darmstadt, Germany) in order to isolate peripheral blood mononuclear cells (PBMCs). Additionally, the layer containing PBMCs or NK cells was isolated washed twice with PBS (Phosphate buffered saline). Then, RNA was extracted from cell pellets [21].

### RNA extraction and cDNA synthesis

Total RNA from WBC pellet was extracted by using TRI reagent (Sigma®, USA) according to manufacturer’s instructions and quantified by using Nanodrop (ND-1000). A 1000 ng of total RNA after quantification was subjected to cDNA synthesis using cDNA synthesis kit (RevertAid First Strand cDNA Synthesis Kit; Thermo Scientific) according to manufacturer’s protocol [20]**.**

### Real-time quantitative reverse transcription-polymerase chain reaction

Quantitative real-time PCR (Applied Biosystems StepOne™ Instrument-Singapore) was used to assess the gene expression levels of NLRC4, NLRP1, ASC, and CASPASE-1 in all subjects using gene-specific primers (Table 1). Expression of the GAPDH gene was used as a reference.

**Table 1:** Primer sequences of NLRC4, NLRP1, ASC, and caspase-1 inflammasomes genes

Gene	Primer	References
Caspase-1	F: GCCTGTTCCTGTGATGTGGAG R: TGCCCACAGACATTCATACAGTTTC	[23]
ASC	F: AGTGGCTGCTGGATGCTCTG R: CATCTTGCTTGGGTTGGTGG	[24]
NLRC4	F: TACACAGCAGGACGAAGACTCAG R: GGCTTCCACAGATGACCCACA	[24]
NLRP1	F: CGCCGCTGGAGAGAAATCTC R: CTCTCTCTTTCTCTGATTTCTGTGT	[25]
GAPDH	F: GCACCGTCAAGGCTGAGAAC R: TGGTGAAGACGCCAGTGGA	[23]

An initial stage of activation at 95 °C for 10 min was followed by 45 cycles of denaturation and annealing (95 °C for 10 s, 65 °C for 15 s, and 72 °C for 20 s). A melt curve appears at the end of the amplification phase. Analysis was done to determine the specificity of the created products [22]. The comparative ΔΔCt method was used for data analysis to achieve relative quantification (RQ), in which the amount of target RNA was normalized to an endogenous reference gene (GAPDH) and compared to a control**.**

### Enzyme-linked immunosorbent assay

A sandwich enzyme-linked immunosorbent assay (ELISA) was used to assess the levels of IL-18 and IL-1β in human serum in accordance with the manufacturer's instructions (Bioassay technology laboratory -Cat. No E0143Hu) [26].

### Detection of ESR, RF, anti-CCP

ESR, RF, and Anti-CCP detection were carried out according the manufacturer's instructions (Globe SCIENTIFIC INC. Sedi-Rate™), (BEACON, RHEUMATOID FACTOR LATEX TEST KIT, (R.A. TEST KIT) J79B), and (Serazym® Anti-CCP IgG), respectively.

### Statistical analysis

Data entry and data analysis were done using SPSS version 22 (Statistical Package for Social Science). Data were presented as number, percentage, mean, standard deviation, median, and range. Chi-square test and Fisher Exact test were used to compare between qualitative variables. In case of parametric data, independent samples *t* test was used to compare quantitative variables between two groups and ANOVA test for more than two groups, while in case of non-parametric data, Mann–Whitney test was used to compare quantitative variables between two groups and Kruskal–Wallis Test for more than two groups. Spearman correlation was done to measure correlation between quantitative variables. P value considered statistically significant when *p* < 0.05.

## Results

### Detection of rheumatoid factor (RF), erythrocyte sedimentation rate (ESR), and anti-cyclic citrullinated peptides (anti-CCP) in all subjects

RF was positive in 32 (86.5%) of treated cases and 10 (66.7%) of untreated cases without statistically significant difference between the two groups, while Anti-CCP was positive in 27 (73.0%) of treated cases and 11 (73.3%) of untreated cases without statistically significant difference between the two groups.

As shown in Table 2, ESR level was significantly higher in the untreated subjects compared with the treated cases. There was no significant difference in RF or Anti-CCP between the two groups.

**Table 2:** Detection of (RF), (ESR), and (Anti-CCP) in all subjects

	Treated (*n* = 37)	Untreated (*n* = 15)	Control (*n* = 25)	*P*-value^1^	*P*-value^2^	*P*-value^3^	*P*-value^4^
Anti-CCP titre(U/ml)							
Mean ± SD	69.46 ± 48.00	53.33 ± 32.84	23.32 ± 2.67	0.000*	0.593	0.000*	0.000*
Median (Range)	66.0 (24.0–160.0)	55.0 (24.0–120.0)	23.0 (19.0–30.0)				
RF titre (IU/ml):							
Mean ± SD	194.19 ± 198.21	96.40 ± 152.49	2.88 ± 1.24	0.000*	0.121	0.000*	0.000*
Median (Range)	80.0 (16.0–512.0)	32.0 (16.0–512.0)	3.0 (1.0–5.0)				
ESR (mm/hr.):							
Mean ± SD	55.19 ± 28.58	66.00 ± 19.01	12.72 ± 2.17	0.000*	0.043*	0.000*	0.000*
Median (Range)	45.0 (15.0–115.0)	60.0 (45.0–115.0)	13.0 (7.0–17.0)				

P1: Comparison among all groups

P2: Comparison between Treated and Untreated groups

P3: Comparison between Treated and Control groups

P4: Comparison between Untreated and Control groups

### Expression of NLRC4, NLRP1, ASC, and CASPASE-1 in PBMC of all subjects

The expression of NLRC4, NLRP1, ASC, and CASPASE-1 in the PBMC among the three groups was statistically significant (*p* value < 0.005), with the untreated RA group having lower expression level of NLRP1 and ASC than the treated and control groups. The expression level of caspase-1 and NLRC4 was significantly higher in the untreated cases compared to treated and healthy control groups. Table 3 shows these results.

**Table 3:** The expression of NLRC4, NLRP1, ASC, and caspase-1 in PBMC of all subjects

	Treated (*n* = 37)	Untreated (*n* = 15)	Control (*n* = 25)	*P*-value^1^	*P*-value^2^	*P*-value^3^	*P*-value^4^
Caspase-1:							
Mean ± SD	1.68 ± 1.30	3.01 ± 1.84	1.04 ± 0.50	0.003*	0.008*	0.183	0.001*
Median (Range)	1.6 (0.10–3.95)	3.3 (0.42–5.84)	1.0 (0.17–2.18)				
NLRC4:							
Mean ± SD	3.63 ± 2.78	4.85 ± 2.62	1.00 ± 0.38	0.000*	0.075	0.000*	0.000*
Median (Range)	3.2 (0.02–9.91)	5.6 (0.75–9.91)	1.0 (0.22–1.87)				
NLRP1:							
Mean ± SD	2.64 ± 1.78	0.62 ± 0.45	2.69 ± 1.55	0.001*	0.000*	0.937	0.000*
Median (Range)	2.28 (0.11–7.48)	0.69 (0.03–1.62)	3.20 (1.00–5.25)				
ASC:							
Mean ± SD	3.96 ± 4.11	0.73 ± 0.52	3.01 ± 1.75	0.011*	0.042*	0.835	0.000*
Median (Range)	2.33 (0.01–12.05)	0.57 (0.04–1.71)	3.53 (0.06–5.42)				

P1: Comparison among all groups

P2: Comparison between Treated and Untreated groups

P3: Comparison between Treated and Control groups

P4: Comparison between Untreated and Control groups

### Serum IL-18 and IL-1β levels

As shown in Table 4, serum levels of both IL-18 and IL-1β in treated subjects were significantly higher (*p* value < 0.005) compared with the treated group.

**Table 4:** Serum levels of IL-18 and IL-1β in all subjects

Serum level (ng/L)	Treated (*n* = 37)	Untreated (*n* = 15)	Control (*n* = 25)	*P*-value^1^	*P*-value^2^	*P*-value^3^	*P*-value^4^
Serum IL-18:							
Mean ± SD	9.46 ± 4.17	12.71 ± 2.50	10.55 ± 2.84	0.012*	0.004*	0.509	0.016*
IL-1 B:							
Mean ± SD	717.55 ± 430.12	1056.17 ± 129.52	820.01 ± 471.15	0.006*	0.001*	0.514	0.040*

P1: Comparison among all groups

P2: Comparison between Treated and Untreated groups

P3: Comparison between Treated and Control groups

P4: Comparison between Untreated and Control groups

### Correlation between the expression of CASPASE-1, NLRC4, NLRP1, and ASC inflammasomes genes

Analysis of the data revealed that there is no meaningful relationship between the expression of the genes for the inflammasomes caspase-1, NLRC4, NLRP1, and ASC among various groups of subjects (*p* value > 0.05). However generally, there was negative correlation between caspase-1 and ASC (*r* = -0.330, *p* = 0.017*).

### Correlation between the expression of CASPASE-1, NLRC4, NLRP1, and ASC inflammasomes genes in relation to serum level of IL-18 and IL-1β

There was no statistically significant correlation between the expression of the genes for the inflammasomes caspase-1, NLRC4, NLRP1, and ASC and serum levels of IL-18 and IL-1β among various groups of subjects (*p* value > 0.05). However generally, there was positive correlation between the expression level of NLRC4 and IL-1β serum level (*r* = value; *p* = 0.286, *p* value = 0.040*).

### Correlation between the expression of caspase-1, NLRC4, NLRP1, and ASC inflammasomes genes to RF titre, anti-CCP titre, ESR in group 1 & 2 (RA cases)

The expression of the genes for the inflammasomes caspase-1, NLRC4, NLRP1, and ASC did not significantly correlate with either RF titre in the treated or untreated groups (*p* value > 0.05). There was a positive correlation between NLRP1 expression level and Anti-CCP titre in the treated group (*r* value = 0.409, *p* value = 0.034*). In addition, a negative correlation between NLRC4 expression level and Anti-CCP in the untreated group was observed (*r* value = − 0.945, *P *value = 0.000*).

Note: The relationship r gauges how strongly two quantitative variables are correlated linearly. Pearson: The constant value of r is an integer between − 1 and 1. *r* > 0 denotes a positive connection. *r* > 0 denotes a negative association.

## Discussion

The current work was initiated to study the involvement of certain inflammasome genes in the pathophysiology of RA in Egyptian patients. In this study, the Rheumatoid arthritis cases were more common in female where 94.6% of treated cases, and 93.3% of untreated cases were females. This is consistent with a previous study reporting a woman-to-man ratio of 1:3 [27]. This distinction is crucial since, in recent decades, gender has attracted much study as a predictor of RA prognosis [28]**.**

In our study, ESR level was significantly higher in the untreated subjects compared with the treated cases. There was no significant difference in RF or Anti-CCP between the two groups.

RA clinical trial inclusion criteria frequently include an ESR of more than 28 mm/h as shown from ESR levels in treated cases and in untreated cases.

Cytokines such as interleukin (IL)-1β and IL-18 are important in autoimmune and inflammatory human diseases. Both cytokines utilize common signal transduction cascades to drive a variety of pro-inflammatory effector networks in various cell types. In tissue and fluid samples isolated from individuals with numerous chronic inflammatory illnesses, IL-1β, IL-18, and other members of the IL-1 superfamily are expressed at high levels [29].The present study showed that the level of serum IL-1β and IL-18 in untreated RA cases was higher compared with treated RA cases and healthy control groups. These results are consistent with a study performed on circulating cytokine levels in patients with rheumatoid arthritis [30] who demonstrated that, if these cytokines are present in measurable amounts prior to treatment, successful treatment with the disease-modifying medication such as sulphasalazine will lead to a reduction in serum levels of IL-lα, IL-1β, and TNF-α.

Inflammasomes play a key role in controlling inflammation. If their expression is dysregulated, they will lead to a number of diseases. Epigenetic pathways are known to be involved in the regulation of inflammasome expression, according to a recent study [31].

In our study, we focused on gene expression of NLRP1, NLRC4, Caspase-1, and ASC inflammasomes to determine whether these inflammasomes included in the pathogenesis of RA through detection of the expression level of them in RA patients and healthy controls as well as to clarify the effect of treatment on their expression through detection of their expression level in treated and untreated cases.

Our findings showed that, the expression levels of NLRP1and ASC inflammasome genes were significantly lower in untreated RA cases compared to treated RA cases and healthy control group. These results are in agreement with previous study made by Wang and his colleagues, and they demonstrated that the expression levels of NLRP1mRNA in PBMC in RA patients were considerably lower than those in the normal control group, indicating that NLRP1 inflammasomes signaling pathways is connected with RA susceptibility [19]. One theory is that there may be an NLRP1 negative regulatory factor that is downregulated in RA inflammatory responses and that inhibits NLRP1 assembly and caspase-1 activation through the NLRP1-signaling pathway [19].

Another interesting finding is that the expression levels of Caspase-1and NLRC4 were higher in untreated cases than treated and control groups. This result supports recently released data that comparing RA patients to healthy controls, caspase-1 was considerably activated. As a protease, caspase-1 catalyzes its substrates to produce biological effects. There are lesser-known pro-IL-1β and pro-IL-18 [32].

Findings of the study proved that there was a statistically significant difference in the expression levels of Caspase-1, NLRP1, and ASC inflammasome genes between the treated group and untreated group. On the other hand, there is no statistically significant difference in the expression level of NLRC4 inflammasome gene. There was a statistically significant difference in the expression levels of Caspase-1, NLRC4, NLRP1, and ASC inflammasomes between untreated RA and healthy control groups while. There was no significant difference between treated RA group and healthy control group in the expression of Caspase-1, NLRP1, and ASC inflammasome genes, but there was a statistically significant difference in the expression level of NLRC4 inflammasome gene.

A study performed by Noroozi and others on the Impact of IFN-β1α treatment on the expression of NLRP3, NLRP1, NLRC4, and AIM2 inflammasomes, as well as caspase-1, IL-1β, and IL-18 as the inflammasomes' downstream molecules; the study revealed that IFN-β1aα modified several inflammasomes' mRNA expression, including NLRP3, NLRC4, and AIM2 (but not NLRP1). Patients receiving IFN-β1α treatment had considerably lower plasma levels of IL-1β [33]. This may explain the change in the level of the expression of inflammasomes in patients received regimen. Addobbati and his assistants mentioned the absence of statistically significant difference between gene expression levels of patients with the disease in remission and patients with high disease activity. Previous finding proved that while the treatment of patients is effective in controlling the disease symptoms, it may not be able to control the deregulation of inflammasome [34].

When we applied correlation, we discovered that the expression of the inflammasome genes caspase-1, NLRC4, NLRP1, and ASC did not significantly correlate across different subject populations. Overall, there was a negative correlation between caspase-1 and ASC.

It was observed that generally, there was no statistically significant relationship between blood levels of IL-18 and IL-1 and the expression of the genes for the inflammasomes caspase-1, NLRC4, NLRP1, and ASC, among different subject groups, while there was positive correlation between the expression level of NLRC4 and IL-1β serum level.

However, there was a positive correlation between NLRP1 expression level and Anti-CCP titre in the treated group and a negative correlation between NLRC4 expression level and Anti-CCP in the untreated group. The expression of the genes for the inflammasomes caspase-1, NLRC4, NLRP1, and ASC did not significantly correlate with either RF titre in the treated or untreated groups.

There was a study done by Yang and his colleagues revealed that activated caspase-1 protein level and serum IL-18 level showed a strong positive connection. However, there was no connection between the level of serum IL-1β and activated caspase-1 protein [35].

Additionally, a study made in China by Wang and others concluded that NLRP1 mRNA expression level showed a negative link with anti-RF antibody that is not consistent with these results, and also showed that ASC, caspase-1 mRNA expression level had no correlation with RF in the RA group that supports our results [19].

Therefore, in addition to the increased level of serum IL-18 and IL-1β, the deregulated activation of these inflammasomes may exacerbate the cell death, contributing to the inflammatory process and its maintenance in RA disease.

## Conclusion and future prospects

The study revealed the role of inflammasomes in the pathogenesis of rheumatoid arthritis which can represent a promising therapeutic target for treating the inflammation in these patients. Our future plan will try to find association between these inflammasomes genes and other common human diseases among Egyptians as cancer, hypertension, type 2 diabetes, and other autoinflammatory diseases.

## Data Availability

The corresponding author can provide the datasets used in and/or analyzed during the current work upon justifiable request.
